# Why Secondary Analyses in Vitamin D Clinical Trials Are Important and How to Improve Vitamin D Clinical Trial Outcome Analyses—A Comment on “Extra-Skeletal Effects of Vitamin D, *Nutrients* 2019, *11*, 1460”

**DOI:** 10.3390/nu11092182

**Published:** 2019-09-11

**Authors:** William B. Grant, Barbara J. Boucher

**Affiliations:** 1 Sunlight, Nutrition, and Health Research Center, P.O. Box 641603, San Francisco, CA 94164-1603, USA; 2 The Blizard Institute, Barts and The London School of Medicine and Dentistry, Queen Mary University of London, London E1 4NS, UK; bboucher@doctors.org.uk

**Keywords:** cancer, D2d, diabetes mellitus, randomized controlled trial, secondary outcomes, supplementation, VITAL, vitamin D

Dear Editor,

The recent paper by Marino and Misra, ‘’Extra-Skeletal Effects of Vitamin D [1]’’, is a heroic effort but fell short on diabetes mellitus type 2 (T2DM) and cancer based on comments regarding two recent large vitamin D randomized controlled trials (RCTs): VITAL for cancer [2]; and D2d for T2DM [3]. They concluded that [2] found no effects of vitamin D supplementation in lowering the incidence of invasive cancer compared to placebo. They concluded from [3] that the benefit of vitamin D in progression to T2DM is likely small. The problem was that they quoted the main finding reported in the abstract but overlooked the secondary analyses.

VITAL enrolled 25,871 participants, of whom 1617 developed cancer, and used 2000 IU/d vitamin D_3_ in the treatment arm. D2d enrolled 2423 participants with prediabetes, 616 of whom progressed to T2DM, and used 4000 IU/d vitamin D_3_ in the treatment arm. These RCTs were published in the *New England Journal of Medicine*, which has a policy of permitting the report of only one major result for any outcome. The fact that both RCTs enrolled large numbers of participants meant that they could obtain results for various subgroups, and, indeed, both papers reported that subgroups had significant or nearly significant reductions in disease outcomes from vitamin D supplementation, as shown in Table 1. (A recent commentary in *Nature* reported that more than 800 scientists have called for the retirement of ‘statistical significance at the *p* = 0.05 level’ in order to “help to halt overconfident claims and to avoid unwarranted declarations of ‘no difference’” [4]). Thus, the findings for these subgroups are important, since, first, they demonstrate that vitamin D supplementation has beneficial effects in reducing risks of both cancer and T2DM, and, second, they identify those most likely to benefit from the vitamin D supplementation used in each RCT. The fact that participants with higher BMI did not benefit is most likely due to the fact that the vitamin D doses were not high enough for them [5]. Ideally, vitamin D RCTs would be based largely on serum 25(OH)D concentrations, not vitamin D doses [6,7,8,9].

Since clinical trials are viewed as the strongest evidence regarding whether any substance, including vitamin D, is effective and has a small risk of adverse effects, it is hoped that future vitamin D RCTs will include an analysis based on serum 25(OH)D concentrations in the protocol and analysis as well as highlight the significant findings of the prespecified secondary analyses.

## Figures and Tables

**Table 1:** Secondary outcomes from two recent large vitamin D randomized controlled trials (RCTs).

Group	Hazard Ratio, 95% Confidence Interval
Cancer incidence	[2]
BMI <25 kg/m^2^	0.76 (0.63 to 0.90)
Blacks	0.77 (0.59 to 1.01)
Cancer mortality rate	
Omit the first year	0.79 (0.63 to 0.99)
Omit the second year	0.75 (0.59 to 0.96)
Prediabetes to diabetes mellitus	[3]
BMI <30 kg/m^2^	0.71 (0.53 to 0.95)
Not taking calcium supplements	0.81 (0.66 to 0.98)
Males	0.82 (0.64 to 1.01)
Age >60.9 yrs	0.80 (0.64 to 1.01)
Non-Hispanics	0.86 (0.72 to 1.02)
